# Induction of immunogenic cell death of tumors by newly synthesized heterocyclic quinone derivative

**DOI:** 10.1371/journal.pone.0173121

**Published:** 2017-03-10

**Authors:** Keum-joo Son, Ki ryung Choi, Chung-Kyu Ryu, Seog Jae Lee, Ho Jeong Kim, Hyunah Lee

**Affiliations:** 1 R&D Center, Pharmicell Co. Ltd., Seongnam-si, Gyeonggi-do, Republic of Korea; 2 College of Pharmacy & Graduate School of Pharmaceutical Sciences, Ewha Womans University, Seodaemun-ku, Seoul, Republic of Korea; 3 Department of Thoracic and Cardiovascular Surgery, Jeju National University School of Medicine, Jeju-si, Jeju-do, Republic of Korea; University of South Alabama Mitchell Cancer Institute, UNITED STATES

## Abstract

Many cancer types are serious diseases causing mortality, and new therapeutics with improved efficacy and safety are required. Immuno-(cell)-therapy is considered as one of the promising therapeutic strategies for curing intractable cancer. In this study, we tested R2016, a newly developed heterocyclic quinone derivative, for induction of immunogenic tumor cell death and as a possible novel immunochemotherapeutic. We studied the anti-cancer effects of R2016 against LLC, a lung cancer cell line and B16F10, a melanoma cell line. LLC (non-immunogenic) and B16F10 (immunogenic) cells were killed by R2016 in dose-dependent manner. R2016 reduced the viability of both LLC and B16F10 tumor cells by inducing apoptosis and necrosis, while it demonstrated no cytotoxicity against normal splenocytes. Expression of immunogenic death markers on the cell surface of R2016 treated tumor cells including calreticulin (CRT) and heat shock proteins (HSPs) was increased along with the induction of their genes. Increased CRT expression correlated with dendritic cell (DC) uptake of dying tumor cells: the proportion of CRT^+^CD11c^+^cells was increased in the R2016-treated group. The gene transcription of Calr3, Hspb1, and Tnfaip6, which are related to immunogenicity induction of dead cells, was up-regulated in the R2016 treated tumor cells. On the other hand, ANGPT1, FGF7, and URGCP gene levels were down-regulated by R2016 treatment. This data suggests that R2016 induced immunogenic tumor cell death, and suggests R2016 as an effective anti-tumor immunochemotherapeutic modality.

## Introduction

Cancer is a serious malady, and in its malignant form, it leads to inevitable death depending on its type and stage of discovery. In many cases, the present anti-cancer therapies with surgical operation, chemotherapy, and radiotherapy cannot adequately therapeutic, as these methods also reveal serious side-effects such as toxicity to normal cells and tissues [1]. To eliminate the tumor completely, inducing tumor specific immunity is considered an effective strategy of therapy [2]. Immunogenic death of tumor cells induced by certain chemotherapeutics like anthracyclines may thus be an effective therapeutic strategy [3,4]. This immunogenic cell death is characterized by the early cell surface exposure of chaperon proteins CRT, HSPs and the late cell apoptosis marker high mobility group box 1 (HMGB1), which affect dendritic cell (DC) maturation and the uptake and presentation of tumor antigens by DCs [5–9]. As such, inducing immunogenic tumor cell death may enhance the effectiveness of DC-based anti-tumor therapies.

**Fig 1:**
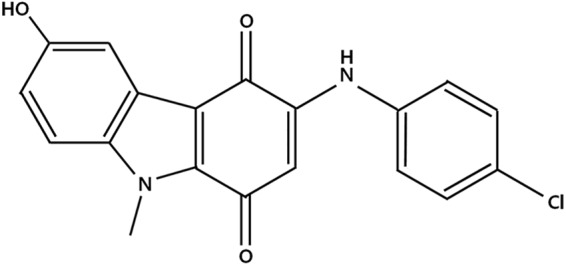
Chemical structure of R2016.

Naturally occurring quinones, which are widely found in plants, animal, fungi and bacteria, possess various potent biological activities including anti-fungal and anti-tumoral activities [10–14]. The cytotoxic effects of these quinones are primarily due to inhibition of DNA intercalation [15]. A variety of analogues of heterocyclic quinone have been designed and synthesized. R2016 (3-(4-chlorophenylamino)-6-hydroxy-9-methyl-9H-carbazole-1,4-dione) (Fig 1) is a newly designed and synthesized heterocyclic quinone compound, and originally devised as an anti-fungal agent [16]. No studies verifying the immunogenic death induction by R2016 as an anti-tumor entity has been reported. In this study, the possibility of R2016 as an immunogenic cell death inducer was tested with the related molecular changes in the target cells. This data may provide the scientific rationale for development of R2016 as a new immuno-chemotherapeutic displaying enhanced anti-tumor potency.

## Materials and methods

### Animals

Pathogen-free female C57BL/6 mice, at 5–6 weeks old, were purchased from the Orient Bio (Seong-nam, South Korea). The mice were provided with water and food *ad libitum* and quarantined under a 12 h light, 12 h dark light cycle in the animal care facility of the Animal Resource Center at the Asan Institute for Life Science and Technology (Asan Medical Center, Seoul, South Korea). Animal care was performed according to the Institute for Laboratory Animal Research (ILAR) guidelines. The mice were acclimated for at least one week before any experiments were conducted. Animal Research was approved by animal research ethics committee in ASAN Medical Center, Seoul, KOREA. (AMC IACUC; approval # 2015-02-185)

### Reagents

R2016 was synthesized and supplied by Dr. Chung-Kyu Ryu (Ewha Women’s University, Seoul, Korea). Doxorubicin hydrochloride was purchased from Sigma-Aldrich (St. Louis, MO, USA). Dulbecco’s modified Eagle’s medium (DMEM) and gentamicin were obtained from GIBCO laboratories (Grand Island, NY, USA) and fetal bovine serum (FBS) was from HyClone Laboratories (Logan, UT, USA). Annexin V/PI and the antibodies for flow cytometric phenotyping were purchased from eBioscience (San Diego, CA, USA); these included the fluorescence labeled-monoclonal Abs against calreticulin (CRT), HSP60, HSP70, and HSP90. ELISA kits for cytokines including TGF-β1, IL-10, and IL-12 were also purchased from eBioscience.

### Cell lines

C57BL/6 syngeneic Lewis lung carcinoma (LLC) and B16F10 (melanoma) cell lines were purchased from the American Type Culture Collection (ATCC) (Rockville, MD, USA). All cell lines were maintained in Dulbecco’s modified Eagle’s medium (DMEM) supplemented with 10% heat-inactivated fetal bovine serum (FBS) and 10 mg/ml gentamicin at 37°C in a 5% CO_2_ atmosphere.

### Cytotoxicity assay

The Cell Counting Kit-8 (CCK-8, Dojindo Laboratories, Kumamoto, Japan) was used to measure the cytotoxicity of R2016 on LLC and B16F10 cells. The cells (2x10^3^ cells/well/200 μl) were cultured in the presence of R2016 (0.1, 0.5, 1, 1.5, 2, 2.5, 3 μg/ml) for 48 h in 96-well plates at 37°C in a 5% CO_2_ incubator. Afterwards a 20 μl CCK-8 solution was added to each well, the plate was re-incubated for 3 h at 37°C and the absorbance at 450 nm was detected using a microplate reader (μ Quant MQX200; Bio-Tek, Winooski, VT, USA).

### Measurement of the viability of spleen cells

Spleen cells were obtained from C57BL/6 mice. Briefly, spleens were disrupted with mechanical force and treated with hypotonic lysis buffer to remove red blood cells. The spleen cells were seeded at a concentration of 1×10^5^ cells/200 μl/well in 96-well culture plates for the cell viability assay. The cells were cultured in presence of R2016 (0.1, 0.5, 1, 1.5, 2, 2.5, 3 μg/ml) for 48, 72 h in 96-well plates at 37°C in a 5% CO_2_ incubator. At the end of each incubation period, the cultured wells were then treated with 20 μl/well of Cell Counting Kit-8 solution (Dojindo Laboratories) for last 3 hr and the optical density of wells was measured at 450 nm using a microplate reader (Bio-Tek).

### Generation of DCs in vitro

Bone marrow (BM) mononuclear cells (MNCs) from tibia and femur of C57BL/6 mouse were isolated and red blood cells (RBC) were eliminated using the RBC lysis buffer (eBioscience). Cells were cultured in RPMI 1640 medium (Gibco) supplemented with 10% FBS (Hyclone), 0.2% gentamycin (10 mg/ml). Purified cells (1×10^6^/ml) were incubated with GM-CSF (1000 unit/ml), SCF (500 unit/ml) and Flt3L (1000 units/ml) at 37°C for 7 days in a humidified CO_2_ incubator for the monocyte expansion and the differentiation. The monocytes were differentiated into DCs by culture in the presence of recombinant IL-4 (1000 U/ml) and GM-CSF (1000 U/ml) for 5–6 days. The above cytokines were from PeproTech (Rocky Hill, NJ, USA).

### Flow cytometric analysis

#### Phenotype observation

The phenotype and ability to induce immunogenicity of R2016-treated cells (LLC, B16F10) were analyzed by direct immunofluorescence staining of cell surface antigens using fluorescein isothiocyanate (FITC) or phycoerythrin (PE) conjugated antibodies against HSP60, HSP70, HSP90, and CRT. Single cells were incubated with fluorescence-labeled surface antibodies in PBS with 0.1% sodium azide and 1% FBS (PBS-CS) for 40 min at 4°C. No more than 1 h after antibody labeling, cells in 300 μl PBS-CS were analyzed with CytoFLEX (Beckman-Coulter, Pasadena, CA, USA). Cultured BM-DC characterization was performed after R2016 treatment by staining with FITC or PE-conjugated mAb against Clec9A, CD197, CD11c, CD8a, CD80, MHC-I, CD86, and MHC-II. Data was analyzed using Flowjo software (Flowjo LLC, Ashland, OR, USA).

#### Apoptosis detection

Cell death was assessed by Annexin V-allo-phycocyanin (APC) staining. R2016-treated tumor cells were collected, washed and re-suspended in an incubation buffer containing Annexin V-APC antibodies. The samples were kept in dark after the addition of the staining antibodies and incubated for 15 minutes before the addition of 0.1% propidium iodide (PI). Labeled cells were observed in a CytoFLEX (Beckman-Coulter, Pasadena, CA, USA). Data were analyzed using Flowjo software.

### Cytokines analysis

The concentration of cytokines secreted by R2016-treated tumor cells was measured using commercial ELISA kits for TGF-β1, IL-10, (eBioscience) and HMGB1 (MyBioSource, Inc., San Diego, CA, USA). To define the R2016-treated cultured-DC character, secretion of IL-12, IFN-γ, IL-10, 1L-6, and TGF-β1 was measured by ELISA kit (eBioscience).

### DC uptake of tumor cells

R2016 treated tumor cells were labeled with CRT-FITC, then co-cultured with CD11c-PE labeled DC for 6 h at 37°C in a humidified CO_2_ incubator. The cells then were washed 2 times. Flow cytometric observation was performed by CytoFLEX (Beckman-Coulter) and the data analyzed by Flowjo software. FITC and PE double positive cells were considered as DCs taken tumor cells.

### Signal protein phosphorylation

The R2016 treated tumor cells were washed once with the Stain Buffer (BD Pharmingen, Franklin Lakes, NJ, USA) and centrifuged to pellet the cells. They were then incubated on ice for 30 min. After washing and centrifugation at 250g for 10 minutes, the supernatants were removed. The cells were resuspended in Stain Buffer at 1x10^7^/ml and aliquoted to 100 μl for each flow tube to continue with PE anti-mouse pSTAT antibody staining (BD Biosciences) and analyzed by flow cytometry.

### Microarray protocol

Total RNA was isolated, labeled, and prepared for hybridization to an 11K mouse oligonucleotide microarray gene chip (Macrogen Inc., Seoul, Korea) following the manufacturer’s instructions. Hybridization was then conducted overnight using 15 μg of labeled RNA product, after which the arrays were scanned using Affymetrix scanners. The gene expression profile of the cells were created using the Affymetrix system (Beyond Bioinformatics ISTECH AATC, Gyeonggi, South Korea) in conjunction with the mouse genome 430A 2.0 Array, which contains approximately 54675 probes. Pre-treatment was conducted using the GCOS global scaling in GenPlex software (Istech Corp., Goyang-si, Gyunggi-do, Korea). Differences in the distribution of data were confirmed by comparing an MA plot of the control array to a plot of the experimental array. Data were considered significant when gene expression changed by at least two-fold at three consecutive time-points when compared to the expression of the control (0 h). Increased gene expression also had to include at least one present call (Affymetrix algorithm) or both control points needed to be present when gene expression increased or decreased.

### RT-PCR

Total RNA was prepared from the R2016 treated-tumor cells using RNeasy mini kit (Qiagen Inc., Germantown, MD, USA). Then RNA was transcribed into complementary deoxyribonucleic acid (cDNA) according to amfiRivert cDNA synthesis master mix kit introduction (Gendepot, Barker, TX, USA). Next, cDNA was used per reaction with TAKARA EX Taq PCR master mix. The RT-PCR reactions were performed on PTC-100 Peltier Thermal Cycler instrument (MJ Research/Bio-Rad, Hercules, CA, USA). The PCR was a 3-stage reaction: initial denaturation at 95°C for 15 minutes, followed by 40 cycles of denaturation at 95°C for 60 seconds, annealing at 60°C for 60 seconds, and extension at 72°C for 60 seconds. Mouse glyceraldehydes-3-phosphate dehydrogenase (GAPDH) acted as an internal reference. The image analysis was performed multi Gauge v3.0. Based on gene sequence published by Genbank database, Primer BLAST software in PUBMED was used to design primer as presented in S1 Table.

### Statistical analysis

The experiments with the same protocol were repeated at least 3 times. Data was expressed as mean±standard error (SE). Statistical significance was determined by ANOVA, followed by Tukey’s range test. P-values of less than 0.05 or 0.001 indicated statistical significance.

## Results

### R2016 induced tumor cell killing

#### Tumor cell death by R2016

**Fig 2:**
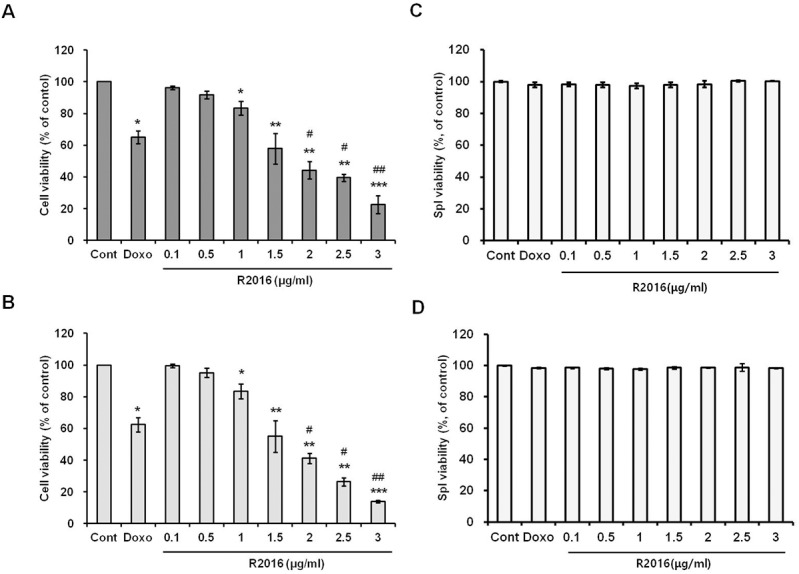
**Effect of R2016 treatment on the viability of tumor cells (LLC (A) and B16F10 (B)) and normal cells (C57BL6 splenocytes, 48 hr (C) and 72 hr (D)).** The tumor cells (2x10^3^ cells/well) and normal cells (1x10^5^ cells/well) were seeded in 96-well plates and treated with different R2016 concentrations (0.1–3 μg/ml) and doxorubicin (0.01 μg/ml). Tumor cells were incubated for 48 hr. Each value is mean±SE of three experiments. Each experiment was done in triplicate. Significant effects compared to untreated-control (*p<0.05 or **p<0.01). ***p<0.005) and doxorubicin treated positive control (#p<0.05, ##p<0.01, ###p<0.005).

Cytotoxicity of R2016 against the lung cancer LLC (Fig 2A), melanoma B16F10 cell lines (Fig 2B) and normal mouse splenocytes (Fig 2C and 2D) was measured. The cells were exposed for 48 hr to different concentrations of R2016 (ranging from 0.1 to 3 μg/ml) (0.28 to 8.52 μmol) or doxorubicin (0.01 μg/ml) (30 nmol) as positive control. R2016 induced tumor cell death (Fig 2A and 2B) without showing cytotoxicity against normal immune cells (Fig 2C and 2D) in a dose-dependent manner. The IC_50_ was about 1.5 μg/ml (4.25 μmol) for both LLC and B16F10 cells (Fig 2A and 2B). Although weaker than doxorubicin, a well-known chemotherapeutic agent, this data confirms the direct tumor cell cytotoxicity of R2016.

#### Analysis of apoptotic cell death of tumor cells

**Fig 3:**
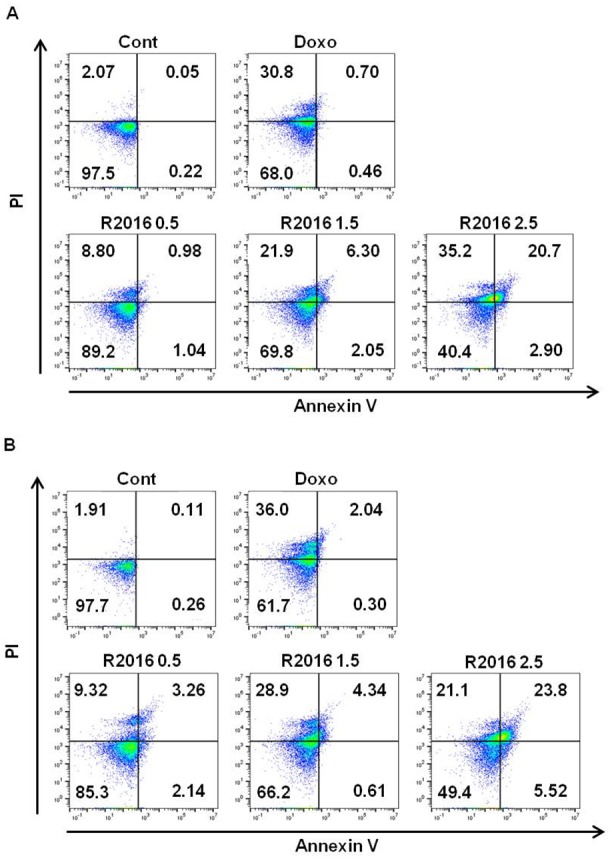
**Apoptotic cell death was observed by R2016 in LLC (A) and B16F10 (B) cell lines.** Cells were treated for 18 hr with the indicated R2016 (0.5, 1.5, 2.5 μg/ml) and doxorubicin (0.01 μg/ml) concentrations. The percentage of apoptotic cells were determined by measuring Annexin V/PI expression using flow cytometry. Each experiment was done in triplicate.

Assays were performed to investigate the type of cell death induced by R2016. In both tumor cell lines, lung cancer and melanoma (Fig 3A and 3B), doxorubicin (0.01 μg/ml) induced rapid necrosis (Annexin V^-^/PI^+^) rather than apoptosis (Annexin V^+^). However, unlike doxorubicin, R2016 was observed to induce the late stage apoptosis (Annexin V^+^/PI^+^) in both the lung cancer (LLC, Fig 3A) and melanoma (B16F10, Fig 3B) cells in a dose-dependent manner.

### R2016 induced immunological death of tumor cells

#### Surface expression of immunogenicity-inducing molecules on the tumor cells treated with R2016

**Fig 4:**
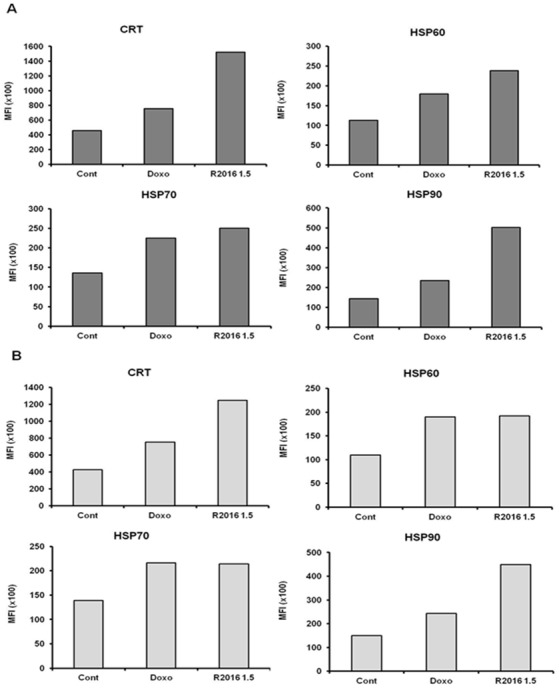
**Expression of DAMPs (CRT, HSP60, HSP70, and HSP90) on the cell surface following R2016 treatment in LLC (A) and B16F10 (B).** Representative histograms of three experiments show the increased expression of CRT, HSP60, HSP70, and HSP90 after R2016 treatment. The expression of the indicated markers is shown as mean fluorescence intensity (MFI).

As immunogenic cell death signal, tumor cell surface expression of calreticulin (CRT) and heat shock proteins (HSP60, HSP70, and HSP90) were measured after R2016 treatment. Flow cytometry results show that at 1.5 μg/ml (4.25 μmol), approximately the IC_50_ dose of R2016, there was a rapid translocation of CRT and heat shock proteins onto the surface of LLC and B16F10 cells 18 hr after the treatment (Fig 4A and 4B). After R2016 treatment, CRT and heat shock proteins expression increased more on the LLC cells than the B16F10 ones. CRT and HSP90 were induced by R2016 more than doxorubicin for both tumor cell types (Fig 4A and 4B).

#### Secretion of *HMGB1*, a nuclear cytokine

**Fig 5:**
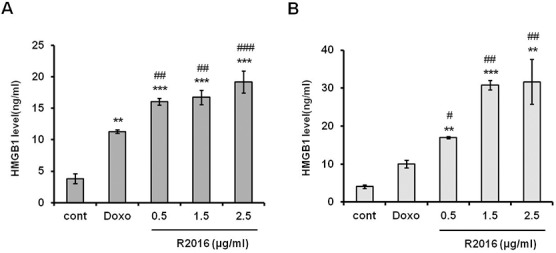
**Secretion of HMGB1 in LLC (A) and B16F10 (B) cell lines. LLC and B16F10 cells were treated with R2016 (0.5, 1.5, 2.5 μg/ml) or doxorubicin (0.01 μg/ml) for 18 hr and cultured supernatants were harvested. HMGB1 protein concentrations in cultured supernatants from LLC and B16F10 cells were measured by ELISA.** Data are presented as mean±SE. Significant effects compared to untreated-control (*p<0.05, **p<0.01, ***p<0.005) and doxorubicin treated positive control (#p<0.05, ##p<0.01, ###p<0.005).

As another immunogenic cell death signal, the levels of HMGB1, a nuclear cytokine that mediates various immune responses [17]. was measured in the supernatant of R2016 treated-tumor cells (Fig 5A and 5B). The HMGB1 released from the R2016-treated cells was significantly increased in a dose-dependent manner compared with that released from the untreated cells. This observed release of HMGB1 was an additional indicator that R2016 induced immunogenic cell death in these tumor cells.

#### Cytokines secretion analysis with ELISA

**Fig 6:**
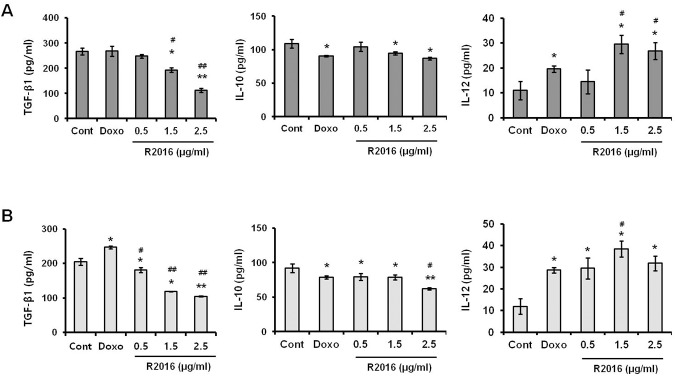
**Effect of R2016 (0.5, 1.5, 2.5 μg/ml) on cytokine secretion from LLC (A) and B16F10 (B) cells. Each value is mean±SE of three experiments.** Each experiment was done in triplicate. Significant effects versus control are indicated with asterisks (*p<0.05, **P<0.01, ***P<0.005) doxorubicin treated positive control (#p<0.05, ##p<0.01, ###p<0.005).

The effect of R2016 on secretion of TGF-β1, IL-10, and IL-12 from the tumor cells was investigated (Fig 6A and 6B). In R2016 treated tumor cell lines, secretion of TGF-β1, an immune-suppressive cytokine, was reduced in dose-dependent manner. The R2016 effect on the IL-10 secretion was however not significant. Normally, it is not usual to detect IL-12 secretion, a Th1 response inducer, from the tumor cells studied, and in this study also, only a negligible amount (≤ 10 pg/ml) of IL-12 was observed from the untreated LLC or B16F10 cells (Fig 6A and 6B). However, interestingly, tumor cells treated with R2016 secreted IL-12 at a detectable level (>30 pg/ml)). The data indicates that R2016 treatment alters the tumor cell microenvironment favorable to anti-tumor immune responses.

#### Uptake of CRT-expressing tumor cells by DCs

**Fig 7:**
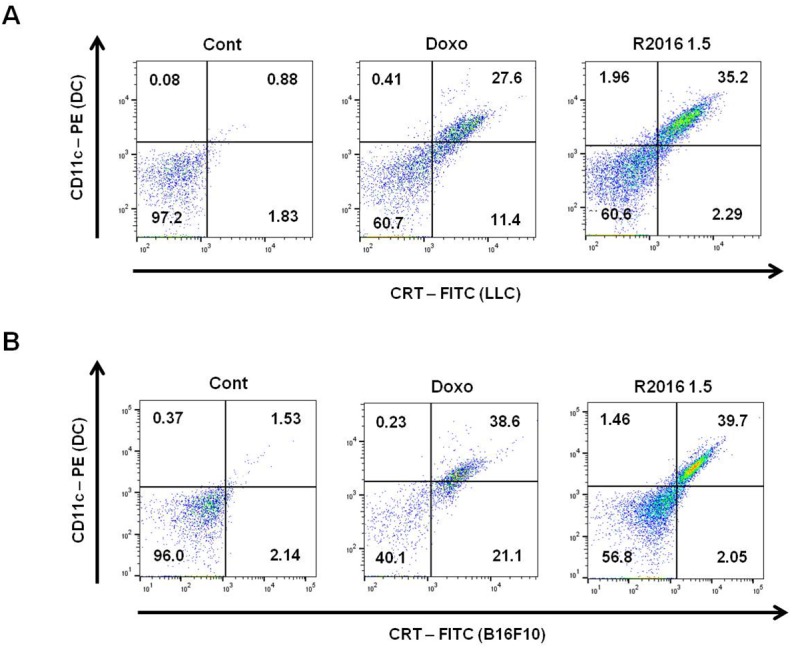
**LLC (A) and B16F10 (B) cells were incubated 18 hr with or without R2016 or doxorubicin at 37°C. LLC and B16F10 cells were then stained with CRT-FITC.** Ex vivo cultured DCs were labeled with CD11c-PE, then co-cultured with CRT-FITC labeled LLC and B16F10 cells for 6 hr. FITC and PE double stained cells were considered as DCs having taken up tumor cells.

CRT is known as an “eat-me” signal, inducing DC uptake of cells with CRT surface expression. It was hypothesized that R2016 would induce the immunogenic cell death of LLC and B16F10 cells with CRT expression. To confirm that R2016 induced immunogenic cell death, DC (CD11c+) and R2016-treated tumor cells (CRT+) were co-cultured. By measuring CD11c+CRT+ double positive cells by flow cytometer, DC uptake of tumor cells was determined. DCs took up the R2016-treated tumor cells more than the untreated control tumor cells (0.88% vs. 35.2% for LLC; 1.53% vs. 39.7% for B16F10 untreated tumor cell vs. R2016 treated tumor cells, respectively) (Fig 7A and 7B).

### Molecular signal alteration induced in the R2016 treated tumor cells

#### Signal Transducer and Activator of Transcription *(STAT)* signal activation

**Fig 8:**
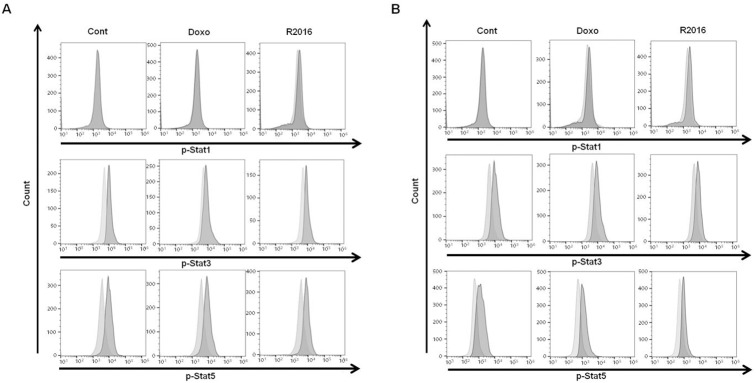
**Phosphorylation of STATs induced by R2016 and doxorubicin in LLC (A) and B16F10 (B) cell lines. Cells were treated for 18 hr with R2016 (1.5 μg/ml) and doxorubicin (0.01 μg/ml).** Then level of intracellular phosphorylated-STAT1 (pSTAT1), pSTAT3 and pSTAT5 were measured by flow cytometry.

STAT intracellular signaling in cancer development, treatment and prognosis has been demonstrated to be significant. The reports indicate that constitutive STAT3 activation is associated with anti-apoptotic as well as proliferative effects in various human cancers. Not surprisingly, poor prognosis and promotion of oncogenesis were reported from a constitutive activation of STAT3 [18–20]. For R2016-treated tumor cells, a dose-dependent reduction of phosphorylated-STAT3 was observed in both LLC and B16F10 cells (Fig 8A and 8B). Also, a constitutive presence of activated (phosphorylated) STAT5 has been reported in cancer cells [21], suggesting that a role in cancer formation and malignant transformation. By R2016 treatment, the levels of phosphorylated-STAT5 were reduced in LLC and B16F10 cells (Fig 8A and 8B). Unlike the STAT 3 and 5 signals which are related to cell proliferation and death, another STAT molecule, namely STAT1, instead has a role in IFN-γ type I and II signaling. R2016 did not affect STAT1 phosphorylation (Fig 8A and 8B).

### RT-PCR analysis

**Fig 9:**
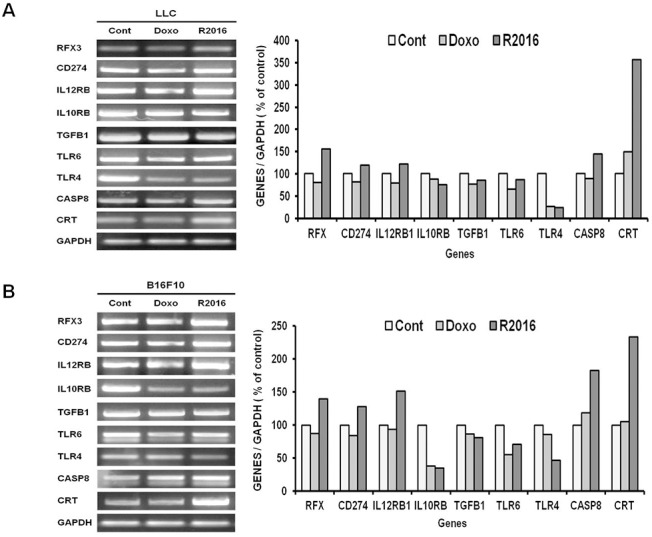
**Gene expression analysis by RT-PCR in LLC (A) and B16F10 (B) cell lines. GADPH was used as the loading control.** Cells were treated for 6 hr with R2016 (1.5 μg/ml) and doxorubicin (0.01 μg/ml).

To confirm the genetic modulation induced by R2016, RT-PCR was performed for differentially expressed genes observed in the microarray data (S2 and S3 Tables). In the R2016 treated B16F10, the expression of IL10RB, TGFB1, and TLR6 were reduced significantly, but not in the LLC cells (Fig 9A and 9B). While, TLR4 expression was reduced significantly by R2016 in both tumor cell types (4x vs. 2x reduction than control in LLC vs. B16F10 cells, respectively). The expression of RTX, CD274, IL12RB1, CASP8, and CRT tended to increase in both LLC and B16F10 cells with R2016 treatment. Among the up-regulated genes, CRT expression was the most significant (3x vs. 2x induction than control in LLC vs. B16F10 cells, respectively) (Fig 9A and 9B).

## Discussion

Heterocyclic quinone compounds have demonstrated potent antifungal and other biologic activities. R2016, a newly synthesized heterocyclic quinone derivative developed by Dr. Chung-Kyu Ryu as an anti-fungal agent, proved in cytotoxic tests to have the ability to kill tumor cells without harming the normal immune cells used. These observations led us to pursue R2016 as a new candidate anti-tumor agent and assay its effects in regards to inducing immunogenic cell death in mouse lung cancer LLC and melanoma B16F10 cells. Both the LLC and B16F10 cells were killed by R2016 at over 0.5 μg/ml in a dose-dependent manner (Fig 2). Melanoma cells were more sensitive to R2016-induced cytotoxicity than the lung cancer cells. Immunogenic cell death is known to be initiated by induction of apoptosis. Unlike doxorubicin induction of necrosis, the R2016-led tumor cell death was observed to be from both necrosis and late apoptosis induction. Also, the R2016-induced apoptotic cell death was dose-dependent with increasing dose of the compound (Fig 3A and 3B). This data suggested the possibility of R2016, a heterocyclic quinone compound, being a new type of immunogenic cell death inducer. This is similar to anthracycline derivatives that are known immunogenic cell death inducers, and are also used as chemotherapeutics.

To confirm the immunogenic cell death induced by R2016, several immunological assays, other than direct cytotoxicity with apoptosis assay, were performed. The enhanced immunogenicity of R2016-killed tumor cells was strongly linked to the induction of CRT and HSPs on the surface of the dying tumor cells and the extracellular release of HMGB1. This translocation of CRT or HSPs onto the surface of dying cells is assumed to be a mechanism underlying the increased immunogenicity of the apoptotic tumor cells as this study revealed it for the treated LLC and B16F10 cells during their R2016-induced apoptotic cell death (Fig 4A and 4B). Furthermore, HMGB1, another immunogenic molecule, was released at the time of cell death induction by R2016 (Fig 5A and 5B). High mobility group box 1 (HMGB1) protein, also known as high-mobility group protein 1 (HMG-1) and amphoterin, is a nuclear cytokine, which can form complexes with ligands to enhance the immune response [22]. The above data supported the premise that R2016 could induce immunogenic cell death of the tumor cells.

Altered levels of immunomodulatory cytokines relating to R2016 treatment of cells as part of their roles in induction of immunogenic tumor cell death were also investigated. Tumor microenvironment is controlled, in part, through cytokine milieu modulated by tumor cells. Immune suppressive cytokines, TGF-β1 and IL-10, are representative factors forming a tumor-favorable environment. In R2016-treated tumor cells, the secretion of both TGF-β1 and IL-10 was suppressed, suggesting to contribute to the immunogenic anti-tumor effect of R2016. In addition, the production of IL-12, a Th1 response-inducing immune stimulatory cytokine, was significantly induced by R2016 treatment of the tumor cells. These observations pointed to R2016 treatment modulating the tumor microenvironment favoring an anti-tumor effect.

One marker of immunogenic cell death is CRT, which is known as an “eat me” signal expressed on the surface of the dying tumor cells, leading to recruitment of DCs to engulf the tumor cells, and inducing tumor specific immunity. Induction of the CRT expression was observed in R2016 treated LLC and B16F10 tumor cells (Fig 4). To confirm the role of CRT as a DC recruitment signal, R2016 treated tumor cells and DCs were co-cultured to observe any differences in the uptake of tumor cells by DCs. The results showed that CD11c^+^ DCs take up the R2016-treated CRT^+^ tumor cells more readily than untreated tumor cells (Fig 7).

Molecular alterations were observed to confirm and define the mechanism of R2016 induced immunogenic tumor cell death. Activation of STAT signaling was observed by flow cytometry, measuring phosphorylated-STAT1, 3, and 5 levels. STAT proteins have been known to be major transcriptional mediators of various fundamental function of cells including those for proliferation, apoptosis, differentiation, and immune responses. Constitutive activation (phosphorylation) of STAT 3 and 5 is known to occur in various cancer cells, and is associated with tumor cell proliferation, invasion and survival along with suppression of anti-tumor immunity [23,24]. In the R2016 treated LLC and B16F10 tumor cells, the levels of phosphorylated STAT 3 and 5 were reduced by 40~60% for both (Fig 8A and 8B). R2016 induced apoptotic tumor cell death and anti-tumor favorable immune induction may thus in part be through the inhibition of STAT3 and STAT5 activation.

The transcriptional changes in R2016 treated tumor cells were also analyzed by microarray profiling (S2 and S3 Figs) and confirmed by RT-PCR (Fig 9). Induction of genes for TNFRSF including Fas-associated death domains (FADD, TRADD) and caspase8 and Fadd like apoptosis regulator (CFLAR) with reduction of anti-apoptotic genes such as STAT6 may explain the function of R2016 as a candidate tumoricidal chemotherapeutic. Along with the upregulation of genes relevant to immunogenic cell death including CRT, expression of various immunogenicity-inducing genes were also increased by R2016 treatment; these included those for cytokine receptor IL12RB1, various chemokines (CCL2, etc), MHC molecule expression regulatory factors (RFX) and B7.1 co-stimulatory molecule (CD274). The levels of the immune suppressive cytokine transforming growth factor beta (TGFB) that would otherwise be favorable to a tumor microenvironment were also inhibited in the R2016 treated tumor cells. Genetic alterations as possible molecular mechanisms for R2016-induced immunogenic cell death of tumor cells were also confirmed by RT-PCR. Interestingly, expressions of TLR 4 and 6 were reduced in the R2016 treated tumor cells, indicating the initiating signal from R2016 is quite different from LPS or other ligands for TLR 4 and 6. Further study is being conducted to find the detailed signaling mechanism for R2016 induced immunogenic cell death of tumor cells, although as shown here, R2016 induced apoptotic tumor cell death may in part be through the inhibition of STAT3 and STAT5 phosphorylation.

In conclusion, the data herein indicate that R2016 can induce immunogenic cell death of tumor cells without killing normal lymphocytes. The data suggests that R2016 may be a new chemotherapeutic agent with improved efficacy and safety. Further study is being performed to establish R2016 as a novel cancer chemotherapeutic.

## Supporting information

S1 Table Primer design of RT-PCR. (PDF)

S2 Table Functional classification of differentially expressed genes in LLC. (PDF)

S3 Table Functional classification of differentially expressed genes in B16F10. (PDF)
