# Supplementary material for: Induction of immunogenic cell death of tumors by newly synthesized heterocyclic quinone derivative
Source: PLoS One. 2017 Mar 10;12(3):e0173121. doi: 10.1371/journal.pone.0173121 (PMC5345761; doi:10.1371/journal.pone.0173121)
Supplement: S1 Table — (PDF) [file pone.0173121.s001.pdf]

Genes	GeneName	Forward primer (5' → 3')	Reverse primer (5' → 3')	Temperature (℃)	Genbank Accession
Rfx3	regulatory factor X, 3 (influences HLA class II expression)	5'-GCCCATGCAACAGAAGCAAA-3'	5'-GGACGTCGGGGATGAGAATC-3'	60	NM_011265
Cd274	CD274 antigen (B7-1)	5'-AAGGGAAATGCTGCCCTTCA-3'	5'-TCATGCTCAGAAGTGGCTGG-3'	59.3	NM_021893
Il12rb1	interleukin 12 receptor, beta 1	5'-CTCACCCTTAGGACCCAGGA-3'	5'-CCGGCCTCTTCAGACACATT-3'	58.7	NM_008353
Il10rb	interleukin 10 receptor, beta	5'-AGAGTCAGGGCTGAATTGGC-3'	5'-GTAAGTTGTCCACGGCTCCA-3'	59.7	NM_008349
Tgfb3	transforming growth factor, beta 3	5'-GTCACTGGAGTTGTACGGCA-3'	5'-GAAGTTGGCATGGTAGCCCT-3'	60	NM_009368
Tlr6	toll-like receptor 6	5'-AATGGTACCGTCAGTGCTGG-3'	5'-GTGAGCAACTGGGAGCAGAT-3'	60	NM_011604
Tlr4	toll-like receptor 4	5'-GGTCAGCAAACGCCTTCTTC-3'	5'-TTTTGTCTCCACAGCCACCA-3'	59.7	NM_021297
Casp8	caspase 8	5'-ATGAGCCTCAAAATGGCGGA-3'	5'-CCAGCAGAAAGTCTGCCTCA-3'	60	NM_009812
CRT	calreticulin	5'-CGAGCTTCAAGCCATTCAGC-3'	5'-TGAAGCCATCCACCTTGCAT-3'	59.9	NM_028500
GAPDH	glyceraldehyde-3-phosphate dehydrogenase	5'-GGCGTGAACCACGAGAAGTATAA-3'	5'-CCCTCCACGATGCCAAAGT-3'	57	NM_008084

S1 Table. Primer design of RT-PCR.
