# Supplementary material for: Induction of immunogenic cell death of tumors by newly synthesized heterocyclic quinone derivative
Source: PLoS One. 2017 Mar 10;12(3):e0173121. doi: 10.1371/journal.pone.0173121 (PMC5345761; doi:10.1371/journal.pone.0173121)
Supplement: S2 Table — (PDF) [file pone.0173121.s002.pdf]

Gene Symbol	LLC_R2016/ LLC_Cont.fc	LLC_Doxo/ LLC_Cont.fc	Genbank Accession	GeneName	Chromosome
Akt2	-2.030292	1.10419	NM_001110208	thymoma viral proto-oncogene 2	chr19
Cdkn1a	3.334137	1.291208	NM_007669	cyclin-dependent kinase inhibitor 1A (P21)	chr6
Cish	2.419534	-1.093602	NM_009895	cytokine inducible SH2-containing protein	chr3
Cntf	2.168157	1.379385	NM_170786	ciliary neurotrophic factor	chr9
Ghr	-2.735856	-1.921325	NM_010284	growth hormone receptor	chr20
Il15	2.983048	2.157364	NM_008357	interleukin 15	chr4
Jak1	-2.410116	-1.655833	NM_146145	Janus kinase 1	chr1
Jak2	-3.060234	1.128074	NM_008413	Janus kinase 2	chr9
Jak3	-2.967493	-1.38365	NM_010589	Janus kinase 3	chr19
Myc	-2.309942	-2.582027	NM_010849	myelocytomatosis oncogene	chr8
Pias2	1.333311	-1.232003	NM_001164170	protein inhibitor of activated STAT 2	Chr18
Pik3r3	-2.099545	1.923916	NM_181585	phosphatidylinositol 3 kinase, regulatory subunit, polypeptide 3 (p55)	chr1
Socs2	2.930192	-1.557418	NM_007706	suppressor of cytokine signaling 2	chr12
Stat6	-3.762501	-2.326166	NM_009284	signal transducer and activator of transcription 6	chr12
Tyk2	-2.608026	-1.649379	NM_018793	tyrosine kinase 2	chr19
Rfx2	2.769875	-1.672132	NM_009056	regulatory factor X, 2 (influences HLA class II expression)	chr19
Rfx1	2.487852	1.034525	NM_009055	regulatory factor X, 1 (influences HLA class II expression)	chr19
Rfx3	2.831504	-1.863057	NM_011265	regulatory factor X, 3 (influences HLA class II expression)	chr9
Cd274	2.571199	-1.030565	NM_021893	CD274 antigen	chr9
Il12rb1	2.507673	1.260033	NM_008353	interleukin 12 receptor, beta 1	chr19
Il10rb	-2.186055	-1.576155	NM_008349	interleukin 10 receptor, beta	chr21
Tab1	-2.459215	-1.513442	NM_025609	TGF-beta activated kinase 1/MAP3K7 binding protein 1	chr22
Tgfb3	-3.141441	-1.081801	NM_009368	transforming growth factor, beta 3	chr14
Tnfrsf12a	2.466106	1.892503	NM_013749	tumor necrosis factor receptor superfamily, member 12a	chr16
Tradd	2.16445	1.043845	NM_001033161	TNFRSF1A-associated via death domain	chr16
Foxred1	2.099648	-1.081674	NM_172291	FAD-dependent oxidoreductase domain containing 1	chr11
Fadd	2.124401	1.293331	NM_010175	Fas (TNFRSF6)-associated via death domain	chr11
Cflar	3.051033	1.54687	NM_009805	CASP8 and FADD-like apoptosis regulator	chr2
Foxred2	3.15514	-1.219645	NM_001017983	FAD-dependent oxidoreductase domain containing 2	chr22
Tlr6	-2.930072	-3.030856	NM_011604	toll-like receptor 6	chr4
Tlr4	-2.304294	-1.278888	NM_021297	toll-like receptor 4	chr9
Ccl2	2.554345	-1.310173	NM_011333	chemokine (C-C motif) ligand 2	chr9
Cxcl16	2.656384	1.364963	NM_023158	chemokine (C-X-C motif) ligand 16	chr17
Cx3cl1	2.242412	-1.064751	NM_009142	chemokine (C-X3-C motif) ligand 1	chr16
Cxcl10	3.358864	-2.525679	NM_021274	chemokine (C-X-C motif) ligand 10	chr4
Cxcl11	2.08537	1.07388	NM_019494	chemokine (C-X-C motif) ligand 11	chr4
Ccl19	3.651656	1.233631	NM_011888	chemokine (C-C motif) ligand 19	chr9
Casp3	1.004281	1.526122	NM_009810	caspase 3	chr4
Casp8	-1.062625	1.147706	NM_009812	caspase 8	chr2
CRT	3.485357	1.165586	NM_028500	calreticulin 3	chr19

S2 Table. Functional classification of differentially expressed genes in LLC.
